# Survival analysis of patients with tuberculosis in Erbil, Iraqi Kurdistan region

**DOI:** 10.1186/s12879-019-4544-8

**Published:** 2019-10-21

**Authors:** Salah Tofik Jalal Balaky, Ahang Hasan Mawlood, Nazar P. Shabila

**Affiliations:** 1 Department of Medical Microbiology, College of Health Sciences, Hawler Medical University, Erbil, Iraq; 2 Department of Community Medicine, Hawler Medical University, Erbil, Iraq

**Keywords:** Tuberculosis, Mortality, Survival analysis, Extrapulmonary, Iraq

## Abstract

**Background:**

Tuberculosis is an important health concern in Iraq, but limited research has examined the quality of tuberculosis care and the survival of the patients. This study aimed to assess the 12-month survival of tuberculosis patients and evaluate the effect of the associated risk factors on patients’ survival.

**Methods:**

We reviewed the records of 728 patients with tuberculosis who were registered and treated at the Chest and Respiratory Disease Center in Erbil, Iraqi Kurdistan Region, from January 2012 to December 2017. Demographic data, the site of the disease, and treatment outcomes were retrieved from patients’ records. Data analysis included the use of the Kaplan–Meier method and the log-rank test to calculate the estimates of the survival and assess the differences in the survival among the patients. The Cox regression model was used for univariate and multivariate analysis.

**Results:**

The mean period of the follow-up of the patients was 7.6 months. Of 728 patients with tuberculosis, 50 (6.9%) had died. The 12-month survival rate of our study was 93.1%. A statistically significant difference was detected in the survival curves of different age groups (*P* < 0.001) and the site of the disease (*P* = 0.012). In multivariate analysis, lower survival rates were only observed among patients aged ≥65 years (hazard ratio = 9.36, 95% CI 2.14–40.95) and patients with extrapulmonary disease (hazard ratio = 2.61, 95% CI 1.30–5.27).

**Conclusion:**

The 12-month survival rate of tuberculosis patients managed at the Chest and Respiratory Disease Center in Erbil was similar to the international rates. The high rates of extrapulmonary tuberculosis and the low survival rate necessitate further studies and action with a possible revision to the tuberculosis management strategy.

## Background

Tuberculosis (TB) is a major global health problem and an important cause of morbidity and mortality in the world. TB is the ninth leading cause of death in the world despite the presence of several treatment strategies to manage the disease [1]. TB is a chronic disease that mostly affects lower socioeconomic classes [2].

TB causes a wide range of clinical infections. The pathogen usually attacks the lungs. However, other body parts are also exposed to the bacterium, such as the spine, kidney, and brain [3]. The central biology of the causative bacterium of the disease, *Mycobacterium tuberculosis*, makes it able to persist in the form of latent TB resulting in long-term asymptomatic infection [4]. Pulmonary TB is usually associated with nonspecific signs and symptoms. Systemic manifestations might include anorexia, fatigue, low-grade fever, night sweats, and weight loss, in addition to cough, which is the most common symptom [5].

*Mycobacterium tuberculosis* is typically transmitted to a human host through the respiratory tract. Therefore, around 80% of TB patients are of pulmonary TB type. However, some pulmonary TB patients present with concomitant extrapulmonary TB, which is a more serious disease. This suggests that the immune systems of pulmonary TB patients who develop concomitant extrapulmonary TB cannot halt *Mycobacterium tuberculosis* bacilli from extending beyond the lung parenchyma [6].

Despite many external factors, death due to TB is still significantly high. According to the World Health Organization (WHO), an estimated 10.4 million people suffered from TB in 2016, while the disease resulted in about 1.3 million deaths among HIV negative cases and 374,000 deaths among HIV positive cases [1]. The Middle East region accounted for 25% of the global burden of TB in 2014. The burden of TB differs by country in the region. The country with the highest incidence in the region is Pakistan (268/100,000 population), while the United Arab Emirates is the country with the lowest value (0.79/100,000 population) [7].

TB is an important public health concern in Iraq. Iraq is a middle burden country with TB, and is ranked 108th globally and 7th in the Eastern Mediterranean region among countries with TB burden size [8]. An estimated 20,000 TB patients were present in Iraq, and around 4000 died from the disease in 2014 [1, 9]. The estimated incidence of TB in Iraq was 43/100000 population with nearly 8268 new and relapse cases reported for 2014 [9]. The prevalence of TB in Iraq is estimated at 74/100000 population, and the mortality is estimated at 3/100000 population [1]. In Sulaimaniyah city, the incidence of all registered TB cases in 2011 was 31/100000 population with around 89% of patients either recovered or completed treatment successfully, and the death rate was about 7% [10].

A major challenge facing most TB programs is a patient that does not complete TB treatment for one reason or another [11], which contributes to the emergence of multidrug-resistant (MDR) TB [12]. The application of the directly observed treatment (DOT) has been related to a worldwide reduction in treatment failure, drug resistance, and relapse [13]. However, the impact of DOT on reducing TB incidence has been inadequate due to non-compliance [14].

Monitoring TB chemotherapy outcome is essential for TB elimination. Even under treatment, more than 10% of patients might die if adherence to treatment is low or if the rates of MDR-TB and HIV infection are high [15]. A study from Cuba reported a death rate of 7% with a significantly higher rate among older patients, men, those coinfected with HIV, and those without a history of incarceration [15]. In a study performed on 202 TB patients, 190 (94.1) had survived through the entire follow-up period, and the mortality rate was 5.9% [16]. Studying the survival patterns of TB patients might assist in determining the risk factors associated with mortality in TB patients. Determining the risk factors associated with death following the diagnosis of TB is crucial to predict prognosis in TB patients and adopt effective interventions to reduce death rates [17].

The national TB program has confirmed a significant rise in TB cases in Iraq over the last few years. This rise was particularly evident in the Kurdistan region of Iraq, mostly due to TB patients fleeing Mosul who had no access to health care in addition to having a large number of displaced people and refugees in remote, hard to reach locations [18]. Few studies on TB deaths have been conducted in Erbil city, and no studies from Erbil have assessed the quality of TB care and the effectiveness of treatment protocols. Therefore, this study aimed to assess the 12-month survival of TB patients and evaluate the effect of the associated unfavorable risk factors on patients’ survival. Knowledge about the survival of TB patients in Erbil city can provide some awareness about the TB situation in the city. It can also direct action toward control programs and improving the quality of TB services by the regional health authorities, including the provision of training and support to TB screening programs as well as early detection and diagnosis of TB cases.

## Methods

This retrospective cohort study was carried out at the Chest and Respiratory Disease Center in Erbil city, Kurdistan Region of Iraq. This center was established in 1978 and has widely expanded with the growth of Erbil population to extend its services to the different districts. The center is primarily an outpatient specialized and consultation clinic. Different types of services are provided in the center, including the diagnosis of TB. Any patient with suspected TB can visit or be referred to the center, and all necessary investigations will be carried out after being seen by a specialist physician. All kinds of medications to treat TB are available, and patients get the course of treatment with monitoring and continuous follow-up.

The records of 728 patients with TB who were registered and treated at Erbil Chest and Respiratory Disease Center from January 2012 to December 2017 were reviewed. The included patients were fully anonymized before accessing their files. The study was approved by the research ethics committee of the College of Health Sciences, Hawler Medical University.

Demographic data, dates of diagnosis and outcome, the site of the disease (pulmonary or extrapulmonary), and treatment outcome were retrieved from patients’ records. For demographic data, only information about the age, gender, and residence of the patients was available on the records. The residence was classified according to the four zones of Erbil city. Zone I includes the area from the center of the city to the 60 m circular street. Zone II includes the area from 60 m circular street to 100 m circular street. Zone III includes the area from 100 m circular street to 120 m circular street. Zone IV includes the area outside 120 m circular street. Thus, Zones I, II, and III are located in more central and crowded areas than Zone IV, which is located in the more open and outer part of Erbil city. The more central zones include old quarters that are characterized by having older housings and infrastructure, and generally accommodate more impoverished people. These factors might affect the prevalence and survival of TB.

The diagnosis of TB was performed based on the National Algorithm for presumptive pulmonary TB [19]; any patient with an unexplained cough for 2–3 weeks or more, in which x-ray and routine diagnostic methods including acid fast bacilli and molecular PCR based method (GeneXpert) were used. Besides, fresh specimens were referred to culture when required. For managing the patients and treatment control, the TB specialist physician made decisions according to the international standard of TB care [20].

For presumptive extrapulmonary TB such as TB meningitis and TB pleurisy, the diagnostic algorithm was different. Appropriate specimens were requested and examined by Xpert MTB/RIF (GenXpert) and TB culture [19]. Depending on weather rifampin-resistant or sensitive, the treatment was started. The final medical diagnosis and prescription of medications are typically performed by TB specialist physicians, according to the International Standards for TB Care [21].

Patients with newly diagnosed pulmonary or extrapulmonary TB are typically provided with a treatment regimen containing 6 months of rifampicin including 2 months of isoniazid, rifampicin, pyrazinamide, and ethambutol and 4 months of isoniazid and rifampicin (2HRZE/4HR) based on the WHO algorithm for treatment of TB patients [1]. After this treatment period, if patients still give positive results, they are given the standardized 2nd line regimen (amikacin, cycloserine, ethionamide, and vitamin B6) or the Category 2 Regimen (streptomycin + HRZE) depending on whether they are MDR or sensitive in GeneXpert Diagnosis [1]. A well-organized program for monitoring, supervision, and support is provided at the center by the International Organization for Migration for all TB patients [18].

Survival time was calculated in months from the date of diagnosis of TB to the date of death or the last visit to the center. Censoring occurred when the patient closed the treatment by cure or complete treatment, treatment failure, or loss to follow-up. Any patient with a positive sputum smear or culture at the start of the treatment but with a negative smear or culture in the last month of treatment and on at least one earlier time was considered a cured case [22]. Any patient who completed treatment and not having a negative sputum smear or culture in the last month of treatment and one earlier time at the minimum was considered a treatment completed case [23]. Any patient with a positive sputum smear or culture at 5 months or later during the treatment was considered a treatment failure [22]. Any patient who failed to attend the center for more than 30 consecutive days after the scheduled date of return to the center was considered a loss to follow-up. Patients, including the loss to follow-up, were actively and routinely contacted by the center to track them and ascertain their outcomes, including mortality. The death was determined by the center through notification and contact with the family or the visit of the family members to the center to obtain a death certificate.

The data were analyzed using the statistical package for the social sciences (version 22). We used the Kaplan–Meier method and the log-rank test to calculate the survival time and compare the survival time among different groups of patients. The statistical significance was set at a *P* value of 0.05. We could not determine the median survival time as fewer than half the patients had died by the end of the observation period. The Cox regression model was used for univariate and multivariate analysis. We calculated hazard ratios and the corresponding 95% confidence intervals (CI) while adjusting for all the available covariates. All covariates significant in the univariate analyses at the *P* = 0.20 level were included for the multivariate model. We also included gender, which we considered a clinically important factor. The proportional hazard assumption was assessed for all covariates, and none of them violated the assumption. We carried out the fit of the multivariable model and used the *P* values from the Wald tests of the individual variables to find out the variables that could be deleted from the model to remove any residual effect. As we only had a limited number of variables, the procedure did not result in removing any of them from the final model.

## Results

**Fig. 1:**
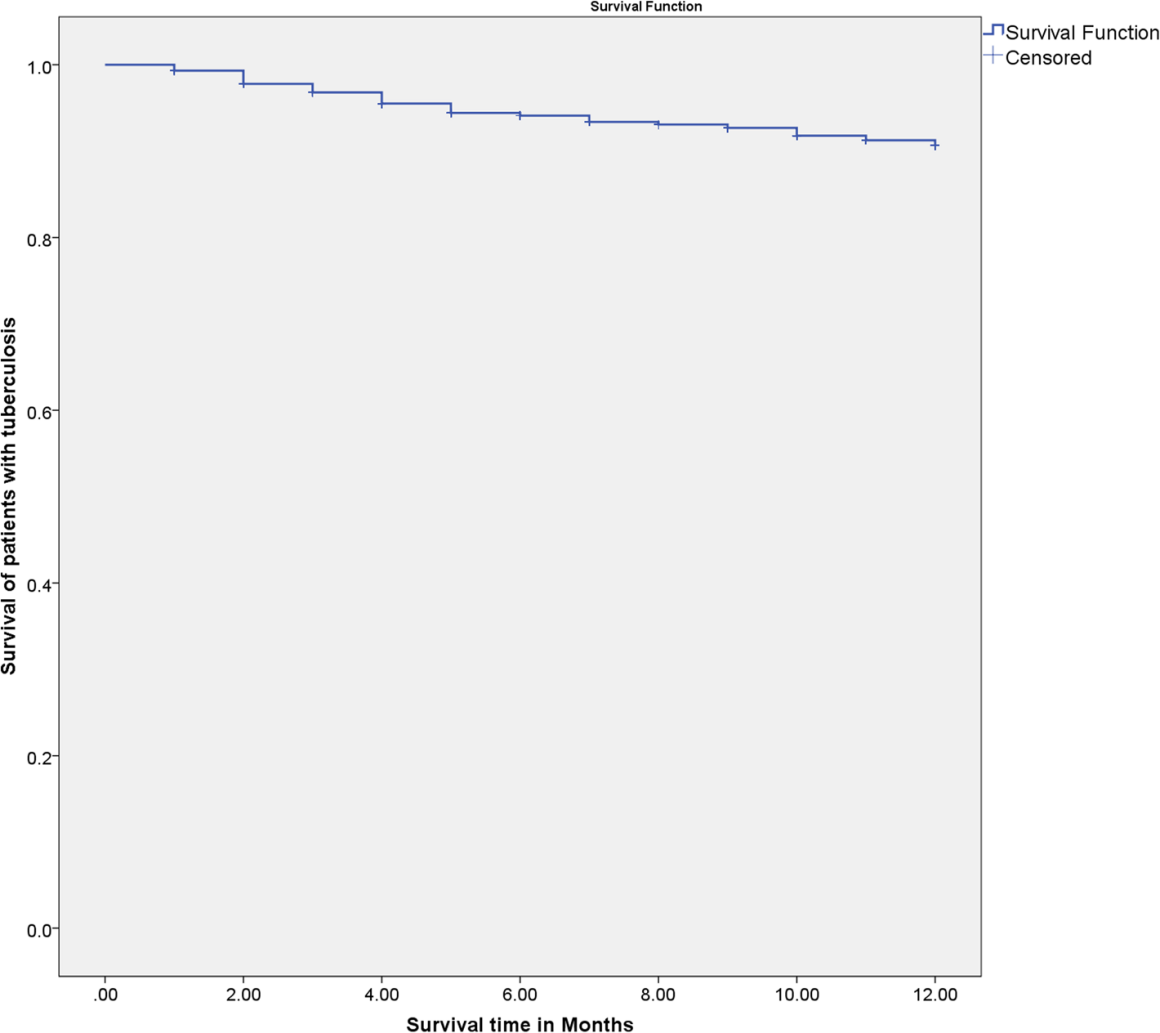
12-month survival rate of tuberculosis patients in Erbil city (2012–2017)

**Fig. 2:**
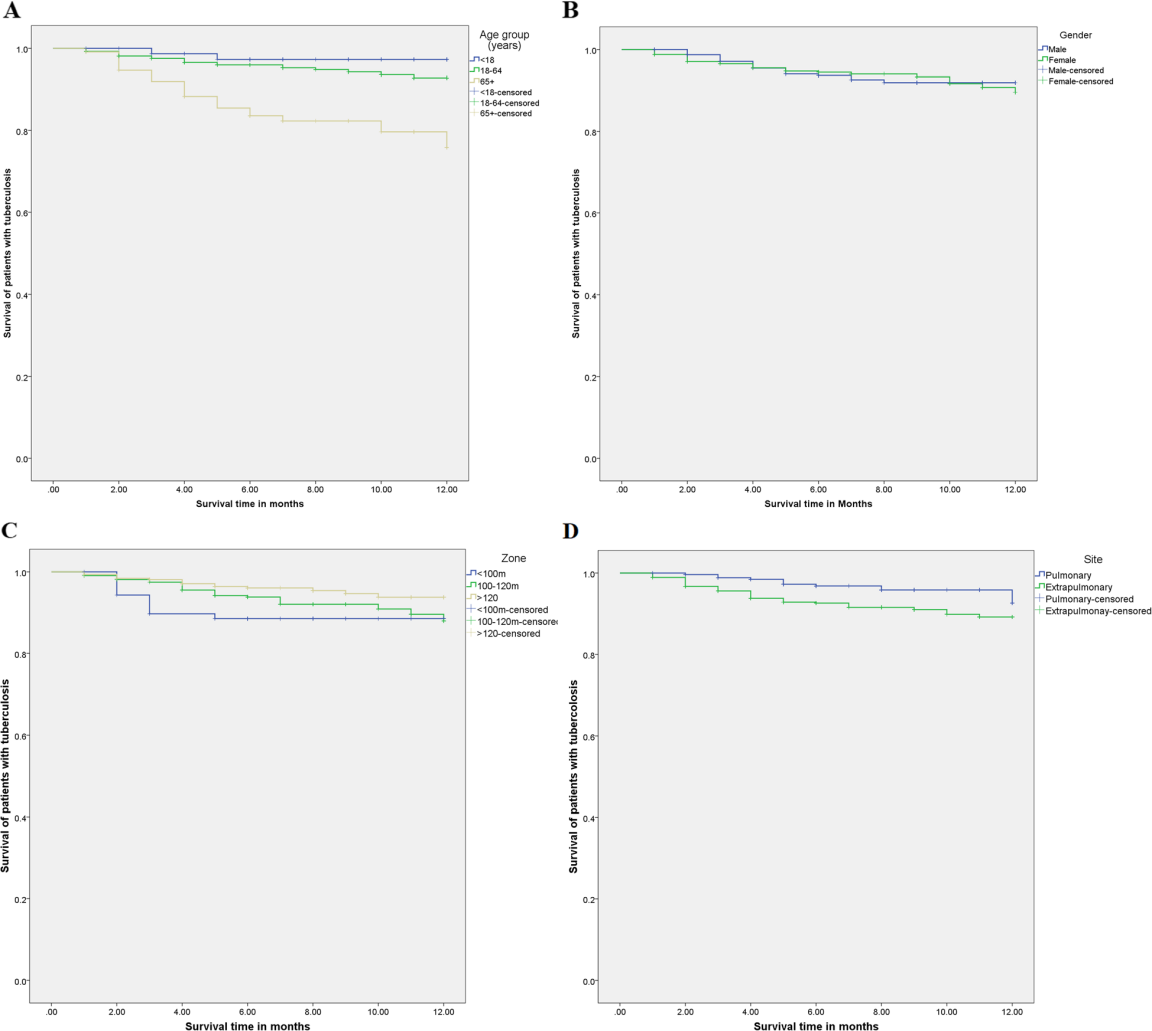
12-month survival rate of tuberculosis patients according to age, gender, zone, and site of the diseases in Erbil city (2012–2017). **a** Survival rate according to age. **b** Survival rate according to gender. **c** Survival rate according to the zone of residence. **d** Survival rate according to the site of the disease

Between January 2012 and December 2017, 728 patients with TB were diagnosed and treated at the Chest and Respiratory Disease Center in Erbil. The age of diagnosis ranged from 3 months to 96 years, with a mean + SD of 40.5 + 20.8 years. Most of the patients (73.6%) were at the age group of 18 to 64 years. A total of 320 patients (44%) were male, and 408 (56%) were female; male to female ratio was 0.79:1. Most of the patients were from Zone III (44%) and Zone IV (43.8%) of Erbil city. Most of the patients had extrapulmonary TB (63.5%), with only 36.5% having pulmonary TB (Table 1). The mean period of the follow-up of the patients was 7.6 months (range, 1–12 months). A total of 133 patients (18.3%) got cured, 422 (58%) had treatment completed, 114 (15.7%) were lost to follow-up, nine (1.2%) had treatment failure, while 50 patients (6.9%) had died. Only three patients were recorded to have MDR-TB, and none of them had died from the disease. The 12-month survival rate was 93.1% (Fig. 1). There was a statistically significant difference in the survival curves of different age groups (*P* > 0.001) and the site of the disease (*P* = 0.012) (Table 1; Fig. 2).

**Table 1:** Demographic and clinical characteristics of patients with tuberculosis in Erbil (2012–2017)

Characteristic	Total sample (=728)		12-month survival				
			Survived (*n* = 678)		Died (*n* = 50)		*P* value
	No.	%	No.	%	No.	%	
Age (years)							
0–17	78	10.7	76	97.4	2	2.6	< 0.001
18–64	536	73.6	509	95.0	27	5.0	
≥ 65	114	15.7	93	81.6	21	18.4	
Gender							
Male	320	44.0	298	93.1	22	6.9	0.995
Female	408	56.0	380	93.1	28	6.9	
Residence							
Zones I and II	89	12.2	79	88.8	10	11.2	0.066
Zone III	320	44.0	295	92.2	25	7.8	
Zone IV	319	43.8	304	95.3	15	4.7	
Primary site							
Pulmonary	266	36.5	256	96.2	10	3.8	0.012
Extrapulmonary	462	63.5	422	91.3	40	8.7	
Outcome							
Cure	133	18.3					
Treatment completed	422	58.0					
Lost to follow-up	114	15.7					
Treatment failure	9	1.2					
Death	50	6.9

In univariate analysis, lower survival rates were observed among patients aged ≥65 years (hazard ratio = 7.96, 95% CI 1.87–33.97) and patients with extrapulmonary disease (hazard ratio = 2.21, 95% CI 1.11–4.42), while higher survival was observed among patients living in Zone IV (hazard ratio = 0.38, 95% CI 0.17–0.85). In multivariate analysis, lower survival rates were only observed among patients aged ≥65 years (hazard ratio = 9.36, 95% CI 2.14–40.95) and patients with the extrapulmonary disease (hazard ratio = 2.61, 95% CI 1.30–5.27) (Table 2).

**Table 2:** Univariate and multivariate analysis of risk factors for survival rates in Erbil (2012–2017)

Characteristics	Univariate analysis				Multivariate analysis			
	*P* value	Hazard ratio	95% CI for Hazard ratio		*P* value	Hazard ratio	95% CI for Hazard ratio	
			Lower	Upper			Lower	Upper
Sex (female)	0.978	0.99	0.57	1.73	0.287	0.73	0.41	1.3
Age (< 18 years)		1				1		
Age (18–64 years)	0.303	2.13	0.51	8.96	0.201	2.57	0.60	10.90
Age (≥65 years)	0.005	7.96	1.87	33.97	0.003	9.36	2.14	40.95
Zone (I and II)		1				1		
Zone (III)	0.270	0.66	0.32	1.38	0.557	0.80	0.38	1.69
Zone (IV)	0.018	0.38	0.17	0.85	0.135	0.53	0.23	1.22
Site (extrapulmonary)	0.025	2.21	1.11	4.42	0.007	2.61	1.30	5.27

## Discussion

In this study, we aimed to assess the survival rate of 728 TB patients registered at the Chest and Respiratory Disease Center in Erbil, Iraq during the period from 2012 to 2017 and assess the effect of associated factors such as the age, gender, site of infection and residence zone on the survival rate. The 12-month survival rate was 93.1%, which was similar to the rates reported in other settings. A study from Sulaimaniyah, Iraqi Kurdistan, reported a survival rate of 93% [10]. Martínez-Rodríguez et al. showed a survival rate of 93% among Cuban patients [15], and Khazaei et al. reported a survival rate of 94.8% in Iran [2].

In the current study, a statistically significant lower survival rate was observed among patients aged ≥65 years (hazard ratio = 9.36, 95% CI 2.14–40.95). Age is a significant risk factor that affects the survival of TB patients, and mortality in TB patients is independently associated with increasing age [24, 25]. Martínez-Rodríguez et al. had observed a higher death rate in patients aged ≥48 years (10.1%) as compared with younger patients (4.1%) [15]. Higher death rates have been noted in elderly patients, which could be related to age-related diseases such as cardiovascular diseases, diabetes mellitus, and malignancy, which are frequently present in older patients with TB [17]. Therefore, it is necessary to screen for these diseases in TB patients and managing them to reduce the risk of death [26]. In addition to age, incomplete treatment is an important risk factor for mortality in TB patients [27].

Our data showed that the frequency of female patients (56%) was slightly higher than male patients (44%). However, a systematic review and meta-analysis study showed a significantly higher TB prevalence among men than women in low- and middle-income countries with a male to female prevalence ratio of 2.21 for bacteriologically positive TB and 2.51 for smear-positive TB. The same study revealed that men are disadvantaged in seeking and accessing TB care in many settings with a male to female ratio for the prevalence-to-notification ratio of 1.55 [28]. Such a disadvantage in seeking and accessing care could be the reason for having a lower frequency of male patients in our study. In the current study, the survival rate was higher in male patients than female patients. However, there was no statistically significant difference between them. Martínez-Rodríguez et al. showed that survival was significantly lower among men (hazard ratio = 1.87) compared to women [15]. A study from Sulaimaniyah, Iraqi Kurdistan, found no significant difference between male and female patients in terms of mortality, although the mortality rate was higher in female patients (8.5%) than male patients (5.3%) [10]. The role of sex hormones such as testosterone and estrogen in immune protection and the pattern of high male mortality due to TB in patients over 40 years old remained unexplained [29].

Our data showed that the frequency of extrapulmonary TB was higher than pulmonary TB (63.5 and 36.5%, respectively). However, a different result had been reported from Iran, as 71.4% of the patients had pulmonary TB [30]. Other studies had found different rates of extrapulmonary TB, including 49.4% in Turkey [31], 39.1% in southern Ethiopia [16], and 21% in the Netherland [32]. Extrapulmonary TB comprises a significant proportion of patients with TB. Factors such as the virulence of the *Mycobacterium tuberculosis* strains, the mode of transmission, and the innate immunity of the host may contribute to differences in the risk of acquiring extrapulmonary TB [33]. However, the high frequency of extrapulmonary TB in our study could directly be related to the fact that the Chest and Respiratory Disease Center is a specialized center that includes more investigation facilities and consultant physicians. There are a number of centers at district levels that provide services for less complicated TB cases, including primarily pulmonary TB. More complicated and difficult to diagnose cases such as extrapulmonary TB are often referred to the Chest and Respiratory Disease Center for diagnosis and treatment. In the current study, a statistically significant lower survival rate was observed in patients with extrapulmonary TB than those with pulmonary TB (hazard ratio = 2.61, 95% CI 1.30–5.27). Another study from Iran showed that the risk of death in patients with extrapulmonary TB was 5.58 times higher than in patients with pulmonary TB [34]. Diagnosis of extrapulmonary TB needs a high clinical suspicion, specific diagnostic procedures, special staining, and culture media for acid-fast bacilli. Delayed diagnosis will result in higher morbidity, mortality, and healthcare cost [2].

The 12-month survival rate was lower in patients living in Zones I, II, and II compared to Zone IV. However, these differences were not significant in multivariate analysis. Zones I, II, and III, located in more central and crowded areas than Zone IV, are characterized by having older housings and infrastructure and accommodate more impoverished people. Other studies have shown that poor housing quality and overcrowding are significantly associated with an increased prevalence of TB [35]. However, limited research has studied the association between TB mortality rate and population residence. Poverty and lack of education can affect the access and utilization of health care services and subsequently affect the outcome of TB [36].

In addition to the factor examined by this study, other factors can be related to the survival of TB patients. For example, the presence of MDR-TB has been associated with elevated rates of treatment failure and relapse, which directly increases the proportions of death amongst these patients [37]. Lower education, previous episodes, diabetes history, and HIV infection were independently associated with mortality in MDR-TB [38].

The findings of this study can help in providing awareness about the TB situation in Erbil city and the Iraqi Kurdistan Region to the regional health authorities, particularly about the management, treatment outcome, and survival of patients with TB. Such awareness can direct action to improve the control programs and the quality of TB services, including training provision and support to TB screening programs, early case finding and diagnosis, and treatment services. The high rates of loss to follow-up and the high rate and low survival of extrapulmonary cases suggest a need for revising and improving the TB management strategy at the center and Iraqi Kurdistan Region. The follow-up system of TB patients can be improved by different methods, such as behavior change communication, contact with patients, text reminders, tracing activities, and application of innovative and effective information systems [39–41]. Earlier diagnosis and complete appropriate treatment can play a major role in improving the outcome of TB treatment and patient survival. Adherence interventions, including incentives and enablers, patient education and counseling, psychological interventions, sending reminders, and effective tracing can help in achieving a complete and appropriate treatment. The provision of adequate training to healthcare providers can also help in enhancing adherence and improving survival [42, 43].

This study has a number of limitations. The generalizability of the findings is limited as the high proportion of extrapulmonary TB suggests that the study population might not be representative of the general TB patients in Erbil city or Iraqi Kurdistan Region. Only a limited number of risk factors were examined, and important known risk factors were not included due to lack of adequate data in the patients’ records at the Erbil Chest and Respiratory Disease Center. The sample size of this study is relatively small for a region with a high burden of the disease and over 5 years as the examined database is only from one center of several centers existing in the Iraqi Kurdistan Region.

## Conclusions

The 12-month survival rate of TB patients managed at Erbil Chest, and Respiratory Disease Center was similar to the international rates. The older age of patients and the presence of extrapulmonary infection constituted significant risk factors and negatively affected the survival rates. The high rates of extrapulmonary infection and the low survival rate among this group necessitate further studies and action with a possible revision to the TB management strategy at the center.

## Data Availability

The datasets used for the current study are available on reasonable request. Please contact the corresponding author, Nazar P. Shabila, for data requests.

## Abbreviations

DOT: Directly observed treatment
HR: Isoniazid and rifampicin
HRZE: Isoniazid, rifampicin, pyrazinamide, and ethambutol
MDR: Multi-drug resistant
TB: Tuberculosis
WHO: World Health Organization

## References

[1] World Health Organization. Global tuberculosis report 2018: WHO/CDS/TB/2018.25. Geneva: WHO; 2018.

[2] Khazaei HA, Rezaei N, Bagheri GR, Dankoub MA, Shahryari K, Tahai A (2005). Epidemiology of tuberculosis in the southeastern Iran. Eur J Epidemiol.

[3] Tavakoli A (2017). Incidence and prevalence of tuberculosis in Iran and neighboring countries. Zahedan J Res Med Sci.

[4] AL-Jebory I, AL-Zaag A, Asmir A (2017). Molecular epidemiology of mycobacterium tuberculosis isolated from pulmonary patients in Iraq. Biol Appl Environ Res.

[5] Al-Kadhimi HM, Dawood HN (2011). The effect of age on clinical and radiological presentation in patients with pulmonary tuberculosis in Baghdad. Iraqi Acad Sci J.

[6] Lin CY, Chen TC, Lu PL, Lai CC, Yang YH, Lin WR (2013). Effects of gender and age on development of concurrent extrapulmonary tuberculosis in patients with pulmonary tuberculosis: a population based study. PLoS One.

[7] Global Health Observatory Data (2018). Tuberculosis.

[8] Durib AK (2018). Prevalence of tuberculosis in Baghdad, Iraq 2012. Int J Sci Res Publ.

[9] Eastern Mediterranean Regional Office, World Health Organization. Iraq tuberculosis http://www.emro.who.int/irq/programmes/tuberculosis.html. Accessed 1 Sept 2019.

[10] Karadakhy K, Othman N, Ibrahimm F, Saeed AA, Amin AA (2016). Tuberculosis in Sulaimaniyah, Iraqi Kurdistan: a detailed analysis of cases registered in treatment centers. Tanaffos.

[11] Rieder HL (2002). Interventions for tuberculosis control and elimination.

[12] Kolappan C, Subramani R, Swaminathan S (2016). Tuberculosis mortality in a rural population from South India. Indian J Tuberc.

[13] Tessema B, Muche A, Bekele A, Reissig D, Emmrich F, Sack U (2009). Treatment outcome of tuberculosis patients at Gondar University Teaching Hospital, Northwest Ethiopia. A five--year retrospective study. BMC Public Health.

[14] Chani K (2010). Factors affecting compliance to tuberculosis treatment in Andara Kavango region Namibia. MA Dissertation.

[15] Martínez-Rodríguez A, González-Díaz A, Armas L, Sánchez L, Martínez-Morales MA, González-Ochoa E (2016). Survival of Cuban patients with pulmonary tuberculosis (2009–2010). MEDICC Rev.

[16] Senbeta A, Weldegerima G, Romha G (2014). Survival analysis and associated risk factors of tuberculosis in-hospital patients’ death in Hawassa city and at Yirgalem town health centers. World J Med Sci.

[17] Pardeshi G (2009). Survival analysis and risk factors for death in tuberculosis patients on directly observed treatment-short course. Indian J Med Sci.

[18] International Organization for Migration-Iraq (2017). UN Migration Agency, Global Fund Support Iraqi Ministry of Health in Combatting TB.

[19] Mohapatra D, Bhatia V (2018). Gene Xpert MTB/RIF: the evidence based path breaking diagnostic tool for tuberculosis diagnosis. Int J Sci Res.

[20] World Health Organization (2014). Guidance for national tuberculosis programmes on the management of tuberculosis in children.

[21] Tuberculosis Coalition for Technical Assistance (2006). International Standards for Tuberculosis Care (ISTC).

[22] Marx FM, Dunbar R, Enarson DA, Beyers N (2012). The rate of sputum smear-positive tuberculosis after treatment default in a high-burden setting: a retrospective cohort study. PLoS One.

[23] Wallis RS, Doherty TM, Onyebujoh P, Vahedi M, Laang H, Olesen O (2009). Biomarkers for tuberculosis disease activity, cure, and relapse. Lancet Infect Dis.

[24] Heunis JC, Kigozi NG, Chikobvu P, Botha S, van Rensburg HD (2017). Risk factors for mortality in TB patients: a 10-year electronic record review in a south African province. BMC Public Health.

[25] Horne DJ, Hubbard R, Narita M, Exarchos A, Park DR, Goss CH (2010). Factors associated with tuberculosis in patients with tuberculosis. BMC Infect Dis.

[26] Hochberg NS, Horsburgh CR (2013). Prevention of tuberculosis in older adults in the United States: obstacles and opportunities. Clin Infect Dis.

[27] Kim L, Moonan PK, Heilig CM, Yelk Woodruff RS, Kammerer JS (2016). Factors associated with recurrent tuberculosis more than 12 months after treatment completion. Int J Tuberc Lung Dis.

[28] Horton KC, MacPherson P, Houben RM, White RG, Corbett EL (2016). Sex differences in tuberculosis burden and notifications in low- and middle-income countries: A systematic review and meta-analysis. PLoS Med.

[29] Blondiaux J, de Broucker A, Colard T, Haque A, Naji S (2015). Tuberculosis and survival in past populations: A paleo-epidemiological appraisal. Tuberculosis.

[30] Marvi A, Asadi-Aliabadi M, Darabi M, Rostami-Maskopaee F, Siamian H, Abedi G (2017). Silent changes of tuberculosis in Iran (2005–2015): A joinpoint regression analysis. J Fam Med Prim Care.

[31] Sunnetcioglu A, Sunnetcioglu M, Binici I, Baran AI, Karahocagil MK, Saydan MR (2015). Comparative analysis of pulmonary and extrapulmonary tuberculosis of 411 cases. Ann Clin Microbiol Antimicrob.

[32] Ohene SA, Bakker MI, Ojo J, Toonstra A, Awudi D, Klatser P (2019). Extra-pulmonary tuberculosis: A retrospective study of patients in Accra, Ghana. PLoS One.

[33] García-Rodríguez JF, Álvarez-Díaz H, Lorenzo-García MV, Mariño-Callejo A, Fernández-Rial Á, Sesma-Sánchez P (2011). Extrapulmonary tuberculosis: epidemiology and risk factors. Enferm Infecc Microbiol Clin.

[34] Rahmanian V, Rahmanian K, Rahmanian N, Rastgoofard MA, Mansoorian E (2018). Survival rate among tuberculosis patients identified in south of Iran, 2005-2016. J Acute Dis.

[35] Cramm JM, Koolman X, Møller V, Nieboer AP (2011). Socio-economic status and self-reported tuberculosis: a multilevel analysis in a low-income township in the eastern cape, South Africa. J Public Health Afr.

[36] Hossain S, Quaiyum MA, Zaman K, Banu S, Husain MA, Islam MA (2012). Socio economic position in TB prevalence and access to services: results from a population prevalence survey and a facility-based survey in Bangladesh. PLoS One.

[37] Santha T, Garg R, Frieden T, Chandrasekaran V, Subramani R, Gopi PG (2002). Risk factors associated with default, failure and death among tuberculosis patients treated in a DOTS programme in Tiruvallur District, South India, 2000. Int J Tuberc Lung Dis.

[38] Chung-Delgado K, Guillen-Bravo S, Revilla-Montag A, Bernabe-Ortiz A (2015). Mortality among MDR-TB cases: comparison with drug-susceptible tuberculosis and associated factors. PLoS One.

[39] Ali SM, Naureen F, Noor A, Fatima I, Viney K, Ishaq M (2018). Loss-to-follow-up and delay to treatment initiation in Pakistan’s national tuberculosis control programme. BMC Public Health.

[40] Ade S, Trébucq A, Harries AD, Ade G, Agodokpessi G, Wachinou P (2015). Follow-up and tracing of tuberculosis patients who fail to attend their scheduled appointments in Cotonou, Benin: a retrospective cohort study. BMC Health Serv Res.

[41] Fraser H, Allen C, Bailey C, Douglas G, Shin S, Blaya J (2007). Information systems for patient follow-up and chronic management of HIV and tuberculosis: a life-saving technology in resource-poor areas. J Med Internet Res.

[42] Bukhary ZA, Alrajhi AA (2007). Tuberculosis treatment outcome in a tertiary care setting. Ann Saudi Med.

[43] Alipanah N, Jarlsberg L, Miller C, Linh NN, Falzon D, Jaramillo E (2018). Adherence interventions and outcomes of tuberculosis treatment: A systematic review and meta-analysis of trials and observational studies. PLoS Med.

